# Scalable Spray Processing Resolves the Dimensionality–Transport Trade‐off in Lead‐Free Indoor Photovoltaics

**DOI:** 10.1002/smtd.71072

**Published:** 2026-09-29

**Authors:** Francesco Lamberti, Remah Elrashedy, Arpita Sarkar, Marcello Righetto, Ribhu Bhatia, Pietro Ostellari, Nicola Dengo, Mariangela Ruggeri, Rosaria Verduci, Andrea Basagni, Carlo Scian, Manas Ranjan Samantaray, Abhisek Chakraborty, Zeynab Skafi, Thomas Meredith Brown, Guilin Liu, Jiacheng He, Zhubing He, Giovanna D'Angelo, Sara Pescetelli, Camilla Ferrante, Simone Meloni, Teresa Gatti, Antonio Agresti

**Affiliations:** ^1^ Department of Chemical Science Università Degli Studi di Padova Padova Italy; ^2^ Department of Information Engineering Università Degli Studi di Padova Padova Italy; ^3^ Zhejiang Beisheng Photovoltaic Co. Ltd. Huzhou Zhejiang China; ^4^ Department of Applied Science and Technology Politecnico Di Torino Torino Italy; ^5^ CHOSE, Centre for Hybrid Organic Solar Energy, Department of Electronic Engineering University of Rome Roma Italy; ^6^ Dept. of Chemical and Pharmaceutical Sciences Università of Ferrara Ferrara Italy; ^7^ Department of Mathematics and Computer Science, Physical Sciences and Earth Sciences University of Messina Messina Italy; ^8^ Department of Physics and Astronomy Università Degli Studi di Padova Padova Italy; ^9^ School of Optoelectronic Information and Physical Science Jiangnan University Wuxi China; ^10^ Department of Materials Science and Engineering, Shenzhen Key Laboratory of Full Spectral Solar Electricity Generation (FSSEG), Institute of Major Scientific Facilities for New Materials Southern University of Science and Technology (SUSTech) Shenzhen China; ^11^ IMM‐CNR Sede Di Messina Messina Italy

**Keywords:** charge transport, dimensional reconstruction, indoor photovoltaics, lead‐free photovoltaics, spray processing

## Abstract

Scalable processing of lead‐free perovskite‐inspired absorbers is often constrained by a fundamental trade‐off: the structural reconstruction needed to improve electronic dimensionality can simultaneously introduce anisotropic textures that hinder vertical charge extraction. Here we show that this trade‐off can be overcome in vacancy‐ordered antimony halides by coupling ultrasonic spray deposition with solvent‐ and halide‐mediated crystallization control. Using Cs_3_Sb_2_I_9_–xCl_x_ as a model system, we developed an ambient‐air deposition process followed by annealing under an N_2_/SbI_3_ atmosphere route based on an amide‐free 2‐methoxyethanol/butanol solvent mixture that yields compact, pinhole‐free films without high‐boiling amide solvents. Chloride incorporation directs annealing‐induced reconstruction from a zero‐dimensional dimeric phase to a layered polymorph, while grazing‐incidence wide‐angle X‐ray scattering reveals that this transformation occurs without generating a strongly detrimental crystallographic texture. Transient absorption spectroscopy further shows suppressed self‐trapping and longer‐lived band‐edge carriers after dimensional reconstruction. As a result, the layered absorber retains an effective out‐of‐plane transport component and enables indoor photovoltaic (iPV) efficiencies up to 6.9% at 1000 lux (6500 K). Our results identify a general route by which scalable spray processing can be used not only to deposit lead‐free absorbers, but also to engineer phase evolution and transport‐relevant texture for efficient vertical optoelectronic operation.

## Graphical abstract

A solvent‐ and halide‐mediated crystallization strategy overcomes the trade‐off between structural reconstruction and vertical charge transport in spray‐deposited Cs_3_Sb_2_I_9_–xCl_x_ absorbers. Controlled phase evolution avoids detrimental crystallographic texture while suppressing self‐trapping and extending carrier lifetimes, enabling efficient vertical transport and indoor photovoltaic efficiencies up to 6.9% at 1000 lux.

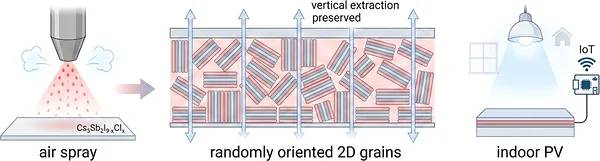

## 1 Introduction

The rapid growth of the Internet of Things, low‐power electronics, and distributed sensing is driving increasing interest in indoor photovoltaic (iPV) technologies capable of efficiently harvesting energy under low‐intensity, narrow‐spectrum artificial illumination [1, 2, 3]. In this context, photovoltaic materials must not only exhibit a suitable bandgap and strong visible‐light absorption, but also meet stringent requirements in terms of environmental safety, long‐term stability, and compatibility with scalable manufacturing [1, 2, 4]. Lead halide perovskites have demonstrated outstanding indoor performance (>44%^3^) [5, 6], yet their intrinsic toxicity, limited operational stability, and frequent reliance on controlled‐atmosphere processing still hinder their translation from laboratory demonstrations to industrial production lines [7, 8]. Perovskite‐inspired materials (PIMs) have therefore emerged as promising alternatives, combining improved environmental compatibility and enhanced stability with optoelectronic properties suitable for indoor energy harvesting [9, 10, 11, 12, 13]. However, replacing Pb(II) with heterovalent, nontoxic cations such as Sb(III) and Bi(III) introduces new challenges related to film formation, crystallization control, and reduced structural and electronic dimensionality [14, 15, 16, 17, 18, 19, 20, 21, 22, 23, 24, 25], which often translate into a trade‐off between improved absorber connectivity and efficient vertical charge extraction. Within this family, Sb‐based compositions such as Cs_3_Sb_2_I_9_ are particularly attractive because of their reduced toxicity, intrinsic stability, earth‐abundant precursors, and optical bandgap well matched to indoor light spectra [26, 27, 28, 29], with recent Cs_3_Sb_2_I_9_‐based devices reaching up to 8.2% at 1000 lux [30]. At the same time, broader pnictogen‐based perovskite‐inspired iPV have already surpassed the 10% threshold [31], making it essential to define the present advance not in terms of absolute record efficiency, but in terms of how scalable processing can be used to achieve competitive performance without compromising vertical transport.

Therefore, their practical exploitation is still limited by two major bottlenecks [27, 32, 33]. First, Cs_3_Sb_2_I_9_ tends to crystallize into a 0D molecular‐like polymorph, which restricts charge‐carrier transport and extraction despite acceptable optical absorption [26, 34, 35]. Second, many of the most effective processing routes reported so far rely on spin coating, inert atmosphere, and high‐boiling or poorly sustainable solvent systems, which are difficult to translate to scalable manufacturing. Overcoming both limitations, namely achieving a transport‐effective phase through an air‐compatible deposition route, is therefore essential for moving this material family beyond proof‐of‐concept iPV demonstrations.

A key strategy to address the structural limitation is chlorine‐mediated dimensional reconstruction. Previous studies have shown that chlorine incorporation can suppress the formation of the transport‐limiting 0D phase and promote the layered 2D polymorph, while also affecting film orientation and crystallinity [30, 36, 37, 38]. Chlorine incorporation is therefore not a novelty factor per se, but rather an established route to improve the structural framework of Sb‐based absorbers. The open challenge is whether this phase control can be integrated into a deposition platform that is compatible with ambient processing and industrial throughput without inducing the texture penalties that often accompany layered reconstruction.

Among scalable coating strategies, ultrasonic spray coating is especially attractive because it enables efficient precursor use, continuous and patternable deposition, and compatibility with air processing, provided that the solvent system is properly engineered [39, 40, 41, 42, 43, 44, 45, 46, 47]. In this regard, solvent choice is critical because it governs droplet drying, film formation, and ultimately the crystallization pathway [44, 45, 46]. For low‐cost, high‐throughput fabrication, however, reliance on high‐boiling, moisture‐sensitive, or toxic solvents remains undesirable [47].

Here, we develop a solvent‐engineering strategy specifically tailored for air‐processed ultrasonic spray coating of Cs_3_Sb_2_I_9_‐based absorbers. Among the screened formulations, a 2‐methoxyethanol/butanol binary mixture provided the best balance between wettability, drying kinetics, and defect suppression, enabling compact and uniform films deposited entirely in air. Post‐deposition annealing then triggered the chlorine‐assisted transformation from the dimeric 0D phase to the layered 2D polymorph. Combined XRD and transient absorption analyses show that this structural evolution sharpens the band edge, suppresses small‐polaron signatures, and prolongs band‐edge carrier persistence, indicating reduced electron–phonon coupling and improved charge transport [9]. Importantly, the layered structure develops without a strongly detrimental crystallographic texture, preserving an effective out‐of‐plane transport component despite the intrinsic anisotropy of layered vacancy‐ordered perovskites [48, 49, 50]. The resulting devices reach up to 6.9% indoor efficiency (stabilized iPCE = 6.5%) at 1000 lux (6500 K) without glovebox processing or high‐boiling amide solvents, placing ambient‐air spray‐coated Cs_3_Sb_2_I_9_‐based devices in a competitive range within Sb‐based indoor absorbers and showing that dimensional reconstruction does not need to come at the expense of out‐of‐plane transport.

## 2 Results and Discussion

The fabrication strategy was designed to control the halide chemistry and phase evolution during film formation and relied on solution processing with a Cs:Sb molar ratio of 3:2, where SbCl_3_ was deliberately introduced as a chloride source to modulate halide coordination [30, 51]. Film deposition was achieved by ultrasonic (US) spray coating performed entirely in ambient air as described in the Supplementary Methods Section, representing a key departure from conventional approaches. A post‐deposition SbI_3_ vapor‐assisted annealing under inert atmosphere was then employed to provide an iodine‐rich reactive environment and promote structural reorganization toward the targeted perovskite framework [26, 51]. The overall processing workflow is schematically summarized in Figure 1a and provides the foundation for the structural, optical, and electronic characterization discussed below. A central objective of this work consisted in identifying a solvent system that simultaneously enables safe handling, reduced toxicity, and scalable air‐processing, preserving sufficient control over crystallization during ultrasonic spray deposition. To this end, four solvent formulations spanning distinct physicochemical profiles were selected to systematically probe solvent‐driven effects on film formation and structural evolution. Acetone/ethanol (Acetone/EtOH) system represents a low‐toxicity, high‐volatility pair that promotes rapid precursor desolvation without requiring antisolvent quenching. In contrast, 1‐Butanol/ethanol (ButOH/EtOH) provides intermediate polarity and slower evaporation kinetics, which is relevant for assessing wet‐film consolidation under milder drying conditions. The ternary water/1‐butanol/ethanol mixture (H_2_O/ButOH/EtOH) introduces a greener component and exploits differential miscibility to modulate nucleation and growth. Finally, 2‐methoxyethanol/1‐butanol (2‐methoxyethanol here referred to as MethoxyEtOH/ButOH) offers a stronger coordination to the metal halide precursors and a higher‐boiling environment expected to enhance precursor solubilization and regulates crystallization on the heated substrate (160°C). Together, these formulations establish a controlled framework to disentangle solvent volatility, coordination strength, and drying kinetics in ambient‐air deposited Cs_3_Sb_2_I_9_ thin films.

**FIGURE 1:**
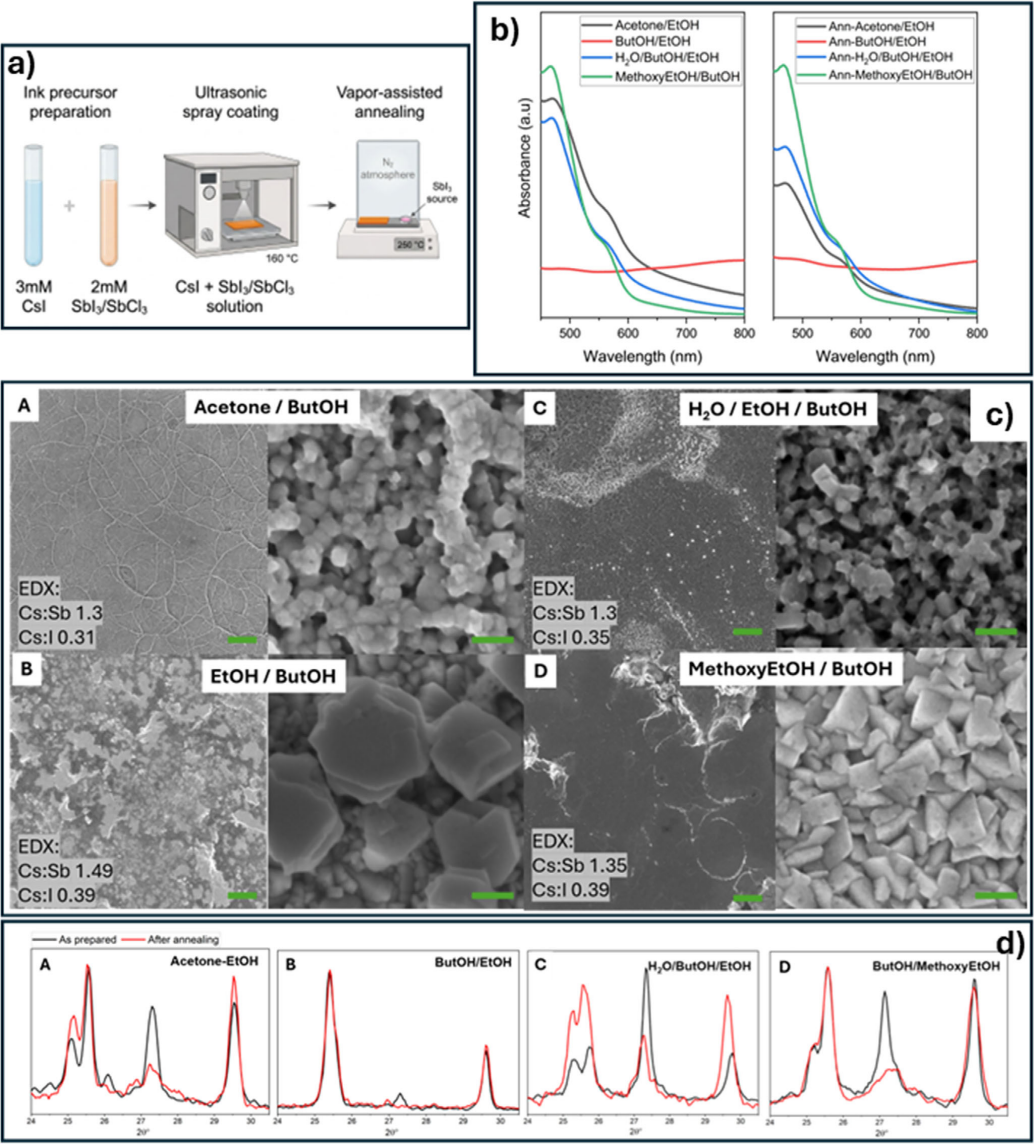
(a) Sketch showing the steps involved in the perovskite thin film fabrication. Created in BioRender. https://BioRender.com/sikqduz; (b) UV–Vis spectra of Cs_3_Sb_2_I_9_ films prepared from different solvent systems (Acetone/EtOH, ButOH/EtOH, H_2_O/ButOH/EtOH, MethoxyEtOH/ButOH) before and after annealing; (c) Top‐view SEM images of Cs_3_Sb_2_I_9_ films deposited from different solvent systems: (A) Acetone/ButOH, (B) EtOH/ButOH, (C) H_2_O/EtOH/ButOH, and (D) MethoxyEtOH/ButOH. Left: low‐magnification (20 µm). Right: high‐magnification (200 nm). EDX‐derived Cs:Sb and Cs:I ratios are reported for each film; (d) XRD patterns of Cs_3_Sb_2_I_9_ films prepared from different solvent systems (Acetone/EtOH, ButOH/EtOH, H_2_O/ButOH/EtOH, ButOH/MethoxyEtOH) recorded as‐prepared (black) and after annealing (red).

Figure 1b shows the optical extinction spectra of Cs_3_Sb_2_I_9_ thin films obtained through ultrasonic spray coating using different solvent mixtures in the as‐prepared state and after thermal annealing. All as‐deposited films exhibit the characteristic absorption onset of Cs_3_Sb_2_I_9_ in the 550–600 nm range; however, pronounced differences in optical density and band‐edge sharpness are observed depending on the solvent system. In particular, films processed from MethoxyEtOH/ButOH and H_2_O/ButOH/EtOH display a more pronounced absorption edge and higher absorbance, indicative of improved film coverage and optical uniformity. By contrast, the ButOH/EtOH formulation results in significantly lower extinction across the visible region, thus suggesting a more limited material deposition and considerable scattering contributions. Notably, a well‐defined spectral feature around ∼500 nm is observed in all samples. Such a feature is consistent with excitonic, or exciton‐related transitions commonly reported in antimony‐based vacancy‐ordered perovskites, where strong carrier localization and reduced dimensionality give rise to distinct optical signatures above the band edge [32]. Upon annealing, the MethoxyEtOH/ButOH formulation yields the cleanest optical response, featuring the sharpest absorption onset and the lowest long‐wavelength background (≥650 nm), indicative of reduced baseline contributions (including scattering) and an improved signal‐to‐noise ratio across the band‐edge region. Significantly, the feature around ∼500 nm remains clearly visible after annealing. The corresponding Tauc plots (Figure S1 of the Supplementary Information), constructed under the assumption of a direct bandgap, yield an optical Eg of 2.06 eV for the as‐prepared MethoxyEtOH/ButOH film, consistent with existing literature [26]. Upon annealing, the absorption onset shows a negligible shift: this outcome suggests that thermal treatment mainly reconfigures the morphology and, hence, the scattering contribution to the extinction spectra, without altering the nature of the electronic transition in the thin film. Overall, this comparison highlights that both solvent formulation and post‐deposition annealing play a crucial role in determining film morphology. Importantly, the degree of film continuity, phase selectivity upon annealing, and band‐edge definition achieved here are comparable to those typically reported for Cs_3_Sb_2_I_9_‐based films deposited via more conventional approaches, such as spin coating with antisolvent treatments [52] and inert‐atmosphere processing [53]. This underscores the effectiveness of the present spray‐based, ambient‐air deposition strategy in achieving analogous material quality through a significantly simplified deposition route.

To directly correlate the optical analysis with film formation, the surface morphology of the resulting Cs_3_Sb_2_I_9_ layers was examined by SEM, supported by semiquantitative EDX analysis to assess local stoichiometry. Qualitatively, the obtained surface morphology shows large differences in coverage, grain connectivity, and crystallite size. Such changes directly correlate with the optical trends observed in Figure 1. Acetone/ButOH (Figure 1c‐A) shows a severely contracted and discontinuous layer characterized by pronounced shrinkage lines at low magnification and, the small, weakly connected grains at nanoscale (200 nm). This fragmented morphology is consistent with the weaker direct transition and the increased scattering tail observed in the UV–Vis spectrum and is further reflected by the low Cs:I ratio (0.31), indicative of complete precursor incorporation. Consistent with the lack of the characteristic onset, EtOH/ButOH (Figure 1c‐B) results in the poorest overall film quality. Both the 20 and 0.2 µm scales show large, isolated faceted crystallites emerging from an irregular and porous matrix, leading to a complete loss of macroscopic continuity. H_2_O/EtOH/ButOH formulation (Figure 1c‐C) shows a partial to a highly heterogeneous film, characterized by extensive uncovered regions interspersed with locally dense areas composed of small, closely packed crystallites. Despite stoichiometric EDX ratios (Cs:Sb = 1.3, Cs:I = 0.35) are close to nominal values, the spatial inhomogeneity limits both optical density and film robustness. In stark contrast, MethoxyEtOH/ButOH formulation (Figure 1c‐D) produces the most homogeneous and compact morphology with densely packed and well‐defined faceting grains. Such an improved microstructure is consistent with the sharper absorption edge observed for this solvent system in UV–Vis measurements. A more continuous and homogeneous film morphology reduces both structural disorder and scattering‐related background contributions, resulting in a better‐defined optical onset. In this context, the EDX‐derived stoichiometric ratios (Cs:Sb = 1.35, Cs:I = 0.39) further support a more complete precursor conversion, which is expected to limit compositional inhomogeneities and associated sub‐bandgap states. Consistent with the morphology and optical quality discussed above, additional UV–Vis ageing experiments (Figure S2 of the SI) show that films processed with the MethoxyEtOH/ButOH formulation exhibit markedly improved optical stability under ambient air exposure compared to the other solvent systems as described in the Supplementary Results dedicated section.

X‐ray diffraction (XRD) was employed to investigate how solvent formulation and post‐deposition annealing influence the crystallographic organization of Cs_3_Sb_2_I_9_ films processed by ultrasonic spray coating. In the Supplementary Information, extended diffractograms are shown for further analysis (Figure S3) for the ButOH/MethoxyEtOH formulation sample. Cs_3_Sb_2_I_9_ exists in two nearly degenerate polymorph families: a 0D dimeric phase (hexagonal, *P63/mmc* space group [28]) and a layered 2D polymorph (trigonal, *P‐3m1* space group [26]) with similar formation energies. While the 0D dimeric phase is arguably more easily synthesized, the formation of the 2D one can be promoted by perturbing the system by compositional tuning and/or thermal treatment, as already reported in the literature [32, 36, 52]. In Figure 1d, the relative amount of the two phases can be qualitatively identified by the presence of the 203 peak (∼27.4°, 1.9 Å^−1^), that is the main peak in the 0D phase, but it's completely absent in the 2D one. The other visible reflections are not uniquely diagnostic, since they are in similar positions in both phases (e.g., around 27°: 006 and 202 peaks for 0D, 003 and 201 for 2D; around 29°: 204 for 0D, 202 for 2D) [27]. The suppression of the intensity of the 0D 203 peak visible from the post‐annealing patterns in Figure 1d clearly indicates that the thermal treatment induces a phase transition toward the 2D phase.

Having selected MethoxyEtOH/ButOH as the best‐performing solvent system, we further refined the 2D/0D phase ratio by introducing chloride anions (see Supplementary Methods) to form Cs_3_Sb_2_I_9‐x_Cl_x_ films. Figure 2a reports the optical absorption spectra of the fabricated Cs_3_Sb_2_I_9_–_x_Cl_x_ films, with increasing nominal chlorine content (*x* = 1, 1.5, 2, 3). Upon chlorine incorporation and post‐deposition annealing, the distinct excitonic feature around ∼500 nm progressively vanishes, consistent with the suppression of strongly localized excitons and the stabilization of a more delocalized, layered electronic structure. While all samples display the characteristic absorption onset of the iodide‐rich Cs_3_Sb_2_I_9_ framework, an overall increase in visible absorption is observed upon chlorine incorporation. Notably, profilometry measurements reveal average film thicknesses of approximately 100, 200, 150, 125, and 110 nm for *x* = 0, 1, 1.5, 2, and 3, respectively, indicating that this trend does not originate from increased film thickness, as chlorine addition slightly reduces the perovskite layer thickness at higher nominal Cl contents. The enhanced absorption is therefore attributed to intrinsic modifications of the optical response rather than to increased material loading. The compact morphology observed by SEM (Figure S4 of the SI) is preserved upon chlorine incorporation, while EDX analysis reveals an improved stoichiometric balance, with both Cs:Sb and Cs:I ratios approaching the nominal value of 1.5 and 0.4 (for *x* = 2), respectively. Together, these observations indicate that chloride incorporation promotes a more complete precursor conversion without altering the microstructural features characteristic of the parent solvent system. Although chlorine is not detected in the final films by SEM–EDX, its role can be rationalized as a transient structural mediator during crystallization. Similar to chloride‐assisted Pb‐based perovskites [54], Cl^−^ is likely to facilitate halide mobility and octahedral reorganization during annealing, lowering the kinetic barrier for the transformation from the dimeric 0D arrangement to the layered 2D polymorph. Once formed, the layered structure remains stabilized even in the absence of measurable chlorine in the final lattice.

**FIGURE 2:**
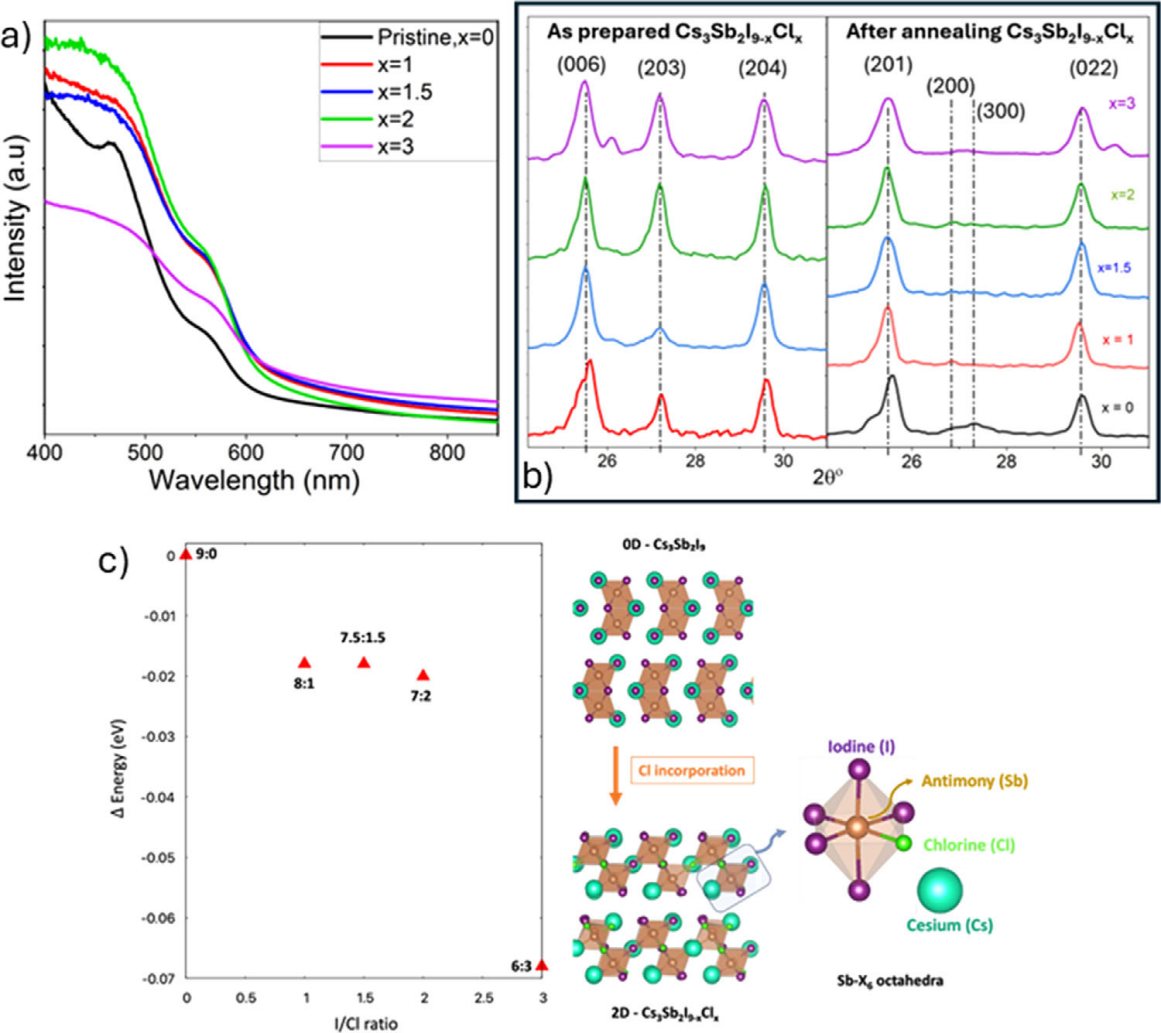
(a) UV–Vis absorption spectra of Cs_3_Sb_2_I_9_–_x_Cl_x_ films (*x* = 0–3) processed using the MethoxyEtOH/ButOH formulation; (b) XRD patterns of Cs_3_Sb_2_I_9_–_x_Cl_x_ films in the as‐prepared state and after annealing, highlighting the suppression of the ≈27.4° region and the emergence of layered‐type reflection regions upon combined Cl incorporation and thermal treatment.; (c) Relative energetics of the 0D and layered (2D) polymorphs of Cs_3_Sb_2_I_9_–_x_Cl_x_ as a function of chloride content. Calculated as the energy difference (ΔE = E^2D^‐ E^0D^), the negative value indicates a thermodynamic driving force for conversion from 0D to 2D phase.

XRD patterns of Cs_3_Sb_2_I_9_–_x_Cl_x_ films before and after annealing (Figure 2b) reveal a progressive transition from a 0D‐phase‐dominated structure to a layered 2D polymorph. Prior to annealing, increasing nominal chloride content does not induce any significant shift in the position of the characteristic 0D‐related reflection, which is consistent with a low degree of chloride incorporation into the as‐deposited perovskite lattice. Upon annealing (right), all chloride‐containing films undergo a pronounced and highly reproducible structural evolution. This transformation is clearly captured by tracking the ≈27.4° reflection, which serves as a reliable fingerprint of the 0D dimeric motif. For all x ≥ 1, this feature is fully suppressed within the detection limits of XRD, and the diffractograms become consistent with a layered structure, displaying the characteristic (201) and (022) reflections of the 2D polymorph. In contrast, the pristine (*x* = 0) film shows only a partial attenuation of the ≈27.4° region upon annealing, underscoring the key role of chloride in facilitating the dimensional reconstruction. These observations are fully consistent with previous Cl‐assisted strategies reported for Cs_3_Sb_2_I_9_‐based absorbers. Several studies [32, 51] have shown that partial substitution of iodide with chloride favors the suppression of the 0D dimeric phase and stabilization of layered Cs_3_Sb_2_I_9–x_Cl_x_ structures, typically under inert processing conditions and often in combination with spin‐coating, antisolvent treatments, and/or A‐site cation engineering. For instance, Li et al. [51]. reported that Cl alloying drives a 0D‐to‐2D transition in Cs_3_Sb_2_I_9–x_Cl_x_ thin films, enabling phase‐pure layered structures suitable for photovoltaics, while Lamminen et al. [32]. demonstrated that small Cl fractions, combined with A‐site cation mixing and glovebox processing, are sufficient to stabilize the layered phase. By contrast, in our ambient‐air deposited, purely inorganic Cs_3_Sb_2_I_9–x_Cl_x_ films deposited by ultrasonic spray coating, chloride incorporation alone does not eliminate the 0D‐associated reflection in the as‐prepared state. A complete transition to the layered phase is achieved only when chloride addition is combined with post‐deposition annealing at 240°C under N_2_. This behavior indicates that chloride acts primarily as an efficient structural mediator of the annealing pathway, rather than as a standalone compositional switch for dimensionality.

Ab initio simulations based on density functional theory (DFT) were performed to investigate the origin of the enhanced stability of the 2D phase of Cs_3_Sb_2_I_9_ relative to the 0D phase upon chlorine incorporation. We computed the 2D–0D relative energy as a function of Cl− content for compositions matching the experimental samples (Figure 2c). In both phases, the SbX_6_ octahedra are distorted rather than perfectly regular. For example, in 2D Cs_3_Sb_2_I_9_ they exhibit two distinct sets of Sb–X bond lengths, with shorter and longer distances of ∼2.9 Å and ∼3.15 Å, respectively [30, 35]. Chlorine can therefore substitute iodine at either site type. We tested substitutions at both short‐ and long‐bond positions and found no significant difference in total energy, indicating no clear site preference for Cl substitution.

Incorporation of chlorine favors the 2D phase over the 0D phase at all concentrations [36]. For intermediate compositions (*x* = *1* and 2), the stabilization is nearly constant at ∼0.02 eV. For *x* = 3 (Cs_3_Sb_2_I_6_Cl_3_), the energetic preference increases markedly to ∼0.07 eV. Although the maximum stabilization is modest (Δ*E* ≈ 0.07 eV for Cs_3_Sb_2_I_6_Cl_3_), its impact on phase populations is amplified through the Boltzmann factor exp(‐Δ*E*/k_B_T), where k_B_T is the thermal energy. At ambient conditions, k_B_T ≈ 0.025 eV, so Δ*E* = 0.07 eV corresponds to an ∼15‐fold increase in the relative stability of the 2D phase compared with the chlorine‐free case. A similar analysis indicates that lower chlorine contents also favors the 2D phase, though more moderately (by approximately a factor of ∼2). These results do not imply that spray‐coating deposition yields exclusively the 2D phase: the crystallized phase depends on both thermodynamics and the kinetics of the crystallization process. Upon annealing, however, the system can approach thermodynamic equilibrium, allowing the more stable phase to emerge and revealing the full potential of chlorine treatment.

Additional mechanistic insight is provided in the Supplementary Information (Figure S5). Ultrafast transient absorption measurements, in agreement with earlier reports on Cs_3_Sb_2_I_9_ thin films [19, 32], reveal that chloride incorporation causes a slight redshift of the ground‐state bleach, strongly reduces the relative amplitude of the photoinduced‐absorption band associated with self‐trapped carriers, and slows the early bleach recovery, indicating reduced small‐polaron formation upon the 0D‐to‐2D transition. This behavior is consistent with the broader role of structural dimensionality in suppressing self‐trapping in perovskite‐inspired absorbers [20, 21, 23, 55]. Complementary DFT calculations further indicate that chloride incorporation improves valence‐level alignment at the Cs_3_Sb_2_I_9__−__x_Cl_x_/Spiro‐OMeTAD interface and lowers the energetic penalty for hole transfer toward the HTM [26, 36, 56, 57].

Having established how solvent formulation, chloride incorporation, and post‐deposition annealing govern the structural and photophysical properties of the Cs_3_Sb_2_I_9_‐based absorbers, their impact on photovoltaic device performance was evaluated under both standard solar (AM1.5G) and indoor illumination conditions. Under AM1.5G illumination, statistical analysis (Figures S8 and S9, Tables S2–S3), reveals a systematic enhancement of all key photovoltaic parameters upon chloride incorporation, with a clear optimum at *x* = 2 for both solvent systems. Devices processed using the MethoxyEtOH/ButOH formulation (see Figure 3a for a SEM cross section image showing the different layer morphology) consistently outperform those based on the ternary solvent route, reaching power conversion efficiency ‐PCE‐ values up to 1.62% (open circuit voltage ‐Voc‐ ≈ 0.66 V, short circuit current density ‐Jsc‐ ≈ 4.92 mA cm^−2^, fill factor ‐FF‐ ≈ 49.7%). In contrast, devices fabricated from the ternary solvent exhibit lower efficiencies (∼1.18%), primarily limited by reduced Voc and Jsc despite comparable or even higher fill factors. This trend mirrors the structural evolution discussed above. The suppression of the 0D‐associated diffraction feature and the stabilization of layered motifs correlate directly with increased Voc, consistent with reduced interfacial recombination and in qualitative agreement with the DFT‐predicted reduction of the interfacial energy barrier. Simultaneously, the enhanced optical density and sharper absorption edge observed for MethoxyEtOH/ButOH films translate into higher photocurrent generation, confirming that morphological compactness and dimensional control are decisive for carrier collection under 1 sun conditions.

**FIGURE 3:**
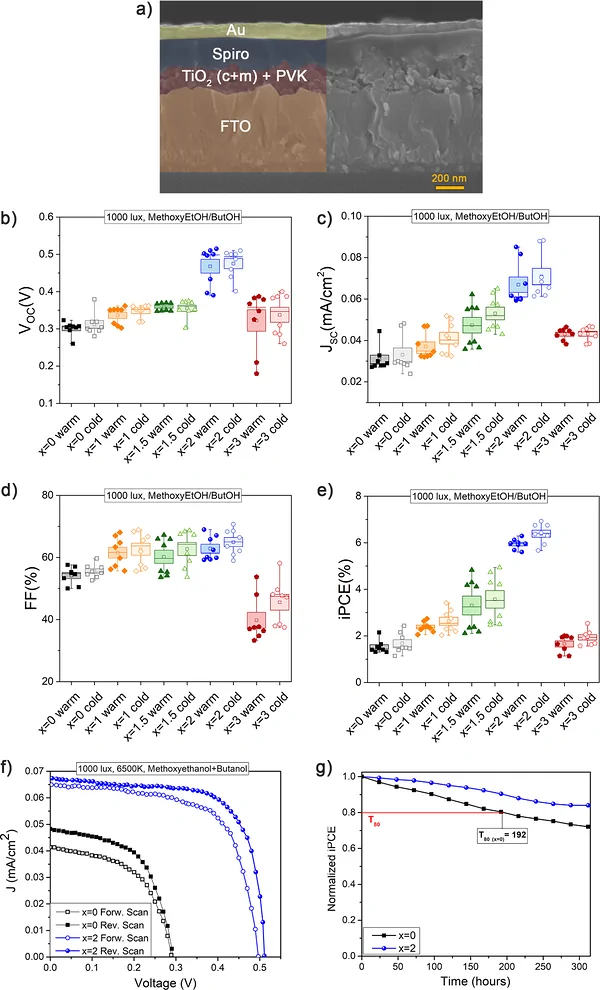
Photovoltaic performance and operational stability of Cs_3_Sb_2_I_9–x_Cl_x_ perovskite‐inspired solar cells processed using the MethoxyEtOH/Butanol formulation under indoor illumination. (a) SEM cross section of the device: PVK refers to the perovskite layer. (b–e) Statistical distributions (n = 8 devices per condition) of b) open‐circuit voltage (Voc), c) short‐circuit current density (Jsc), (d) fill factor (FF), and (e) indoor power conversion efficiency (iPCE) measured at 1000 lux for different chloride contents (*x* = 0–3) and LED color temperature (“warm @ 3000K” and “cold @ 6500K”). Moderate chloride incorporation (*x* = 2) yields the highest and most reproducible device performance. (f) Representative J–V curves of the best‐performing devices (*x* = 0 and *x* = 2) measured at 1000 lux (6500 K), showing forward and reverse scans. (g) Time‐dependent operational cell stability under continuous 1000 lux (6500 K) illumination @ open circuit ‐OC‐ condition, presented as normalized PCE versus time. The *x* = 2 devices exhibit significantly improved stability, maintaining >80% of their initial efficiency beyond 320 h, whereas chlorine‐free devices reach T_80_ at ∼192 h.

The performance differences between solvent systems become even more pronounced under low‐intensity indoor illumination. At 1000 lux (6500 K), a regime particularly relevant for low‐power energy harvesting applications, the MethoxyEtOH/ButOH device with *x* = 2 delivers an iPCE (reverse voltage scan) of 6.96% (stabilized iPCE @ MPP = 6.5%), with a Voc ≈ 0.51 V, Jsc ≈ 67.2 µA cm^−2^, and FF ≈ 68.69% (Figure 3b‐g, Table S4 and Figure S10a), representing a substantial improvement over ternary solvent‐based devices (max iPCE = 3.96% @ 1000 lux, 6500K. See also Figures S11‐S12; Tables S4–S5). This value places the present device among the best‐performing Cs_3_Sb_2_I_9_‐based iPV obtained through an ambient‐air deposition route, while remaining below the current 8.2% benchmark reported for the best‐performing Cs_3_Sb_2_I_9_‐based devices [30]. The performance gain arises primarily from a pronounced increase in Voc (0.30 → 0.50 V) and improved FF, consistent with reduced interfacial barriers and suppressed non‐radiative recombination.

The influence of illumination color temperature was also examined for the MethoxyEtOH/ButOH devices. Cell performance systematically improves moving from warm (3000K) to cold (6500K) LED lamp, showing maximized performance @ 6500 K (Figure 3), which was therefore selected as the standard condition for all subsequent iPV measurements. Importantly, when the illumination intensity is further reduced to 200 lux (6500 K), the optimized devices maintain superior performance, delivering a maximum iPCE of 6.15% (Voc ≈ 0.40 V, Jsc ≈ 19.04 µA cm^−2^, FF ≈ 61.22%, Figure S13), while the best chlorine‐free device achieves 2.97% iPCE, with slightly reduced Voc and FF, confirming the strong suitability of the optimized absorber system for efficient energy harvesting under extremely low indoor illumination levels. The structural stabilization induced by solvent engineering and chloride incorporation is further reflected in operational robustness. Under continuous indoor light soaking at 1000 lux (6500 K) and open circuit ‐OC‐ condition, MethoxyEtOH/ButOH devices with *x* = 2 maintain performance above 80% of their initial efficiency for over 320 h, while chlorine‐free counterparts reach T_80_ after ∼192 h (Figure 3g). This enhanced stability is consistent with the hypothesis that partial dimensional reconstruction and improved film compactness mitigate degradation pathways associated with morphological discontinuities and interfacial defects.

The inferior 1 Sun performance is primarily transport‐limited due to low carrier mobility and higher trap density arising from air spray deposition. However, under low illumination (1000 lux), the device operates in a low‐injection regime where bimolecular recombination and space‐charge effects are strongly suppressed. As a result, the relative PCE significantly improves despite modest 1 Sun performance. The dimensional reorganization and interfacial barrier reduction enable efficient operation in the low‐intensity, narrow‐spectrum conditions typical of indoor environments, positioning these spray‐processed Cs_3_Sb_2_I_9–x_Cl_x_ absorbers as promising candidates for scalable indoor energy harvesting. Taken together, the device results demonstrate that solvent selection, moderate chloride incorporation, and post‐deposition annealing act synergistically to produce a structurally and electronically optimized Cs_3_Sb_2_I_9_ absorber. The MethoxyEtOH/ButOH formulation combined with *x* = 2 chloride consistently yields the most compact morphology, the highest fraction of layered stacking, and the most favorable photophysical signatures across all characterization techniques. The suppression of the 0D‐associated diffraction features and the emergence of the layered (112)/(200) reflections upon Cl + annealing correlate directly with the enhanced Voc and FF, while the increased optical density and sharpened absorption edge translate into higher Jsc under both solar and indoor illumination. Transient Absorption Spectroscopy (TAS) further confirms reduced carrier self‐trapping and enhanced persistence of band‐edge populations in the Cl‐modified films, conditions that are particularly conducive to efficient charge extraction under low‐photon‐flux operation (see Figure S6).

Additional ancillary measurements further support the beneficial effect of chloride incorporation (Figure S14). EIS reveals a markedly reduced semicircle for *x* = 2 devices, indicating lower interfacial charge‐transfer resistance and more efficient carrier transport across the device stack, in line with the improved FF (Figure S14a). Dark J–V curves show suppressed reverse‐bias current and improved diode rectification, consistent with reduced shunt pathways and lower trap‐assisted recombination (Figure S14b), while transient photovoltage measurements evidence longer photovoltage rise time (τ_rise_), particularly in the low‐bias regime relevant to indoor operation (Figure S14c). Together, these results support the view that chloride‐induced dimensional reconstruction mitigates carrier localization and non‐radiative losses, helping explain the pronounced Voc and efficiency gains observed under low‐photon‐flux conditions.

To investigate whether the layered polymorph obtained upon chlorine incorporation develops a crystallographic texture potentially unfavorable for out‐of‐plane charge extraction, we investigated the crystallographic texture of the thin films using grazing‐incidence wide‐angle X‐ray scattering (GIWAXS). While layered vacancy‐ordered antimony halides often suffer from anisotropic charge transport, particularly when the inorganic octahedral layers are preferentially oriented parallel to the substrate [48, 58], our results indicate that the chlorine‐containing films do not develop the strongly preferential texture typically associated with this limitation.

Figure 4 summarizes the structural analysis combining laboratory XRD and synchrotron‐based GIWAXS. The Le Bail refinement of the laboratory XRD patterns (Figure 4a) confirms the progressive structural conversion from the dimeric 0D polymorph to the layered 2D phase upon chlorine incorporation, with the *x* = 2 composition dominated by the 2D structure. Importantly, the lineshape of the overlapping reflections around 25.9° (q ≈ 1.83 Å^−^^1^), arising from the 003 and 10−2 reflections of the layered phase, provides insight into the crystallographic texture of the films [27, 51]. Simulations based on the March–Dollase model [59] (Figure 4b) show that strong preferential orientation along the [001] direction would produce a pronounced distortion of the peak profile. However, comparison with the experimental data indicates that the diffraction line‐shape is consistent with a largely random crystallite distribution.

**FIGURE 4:**
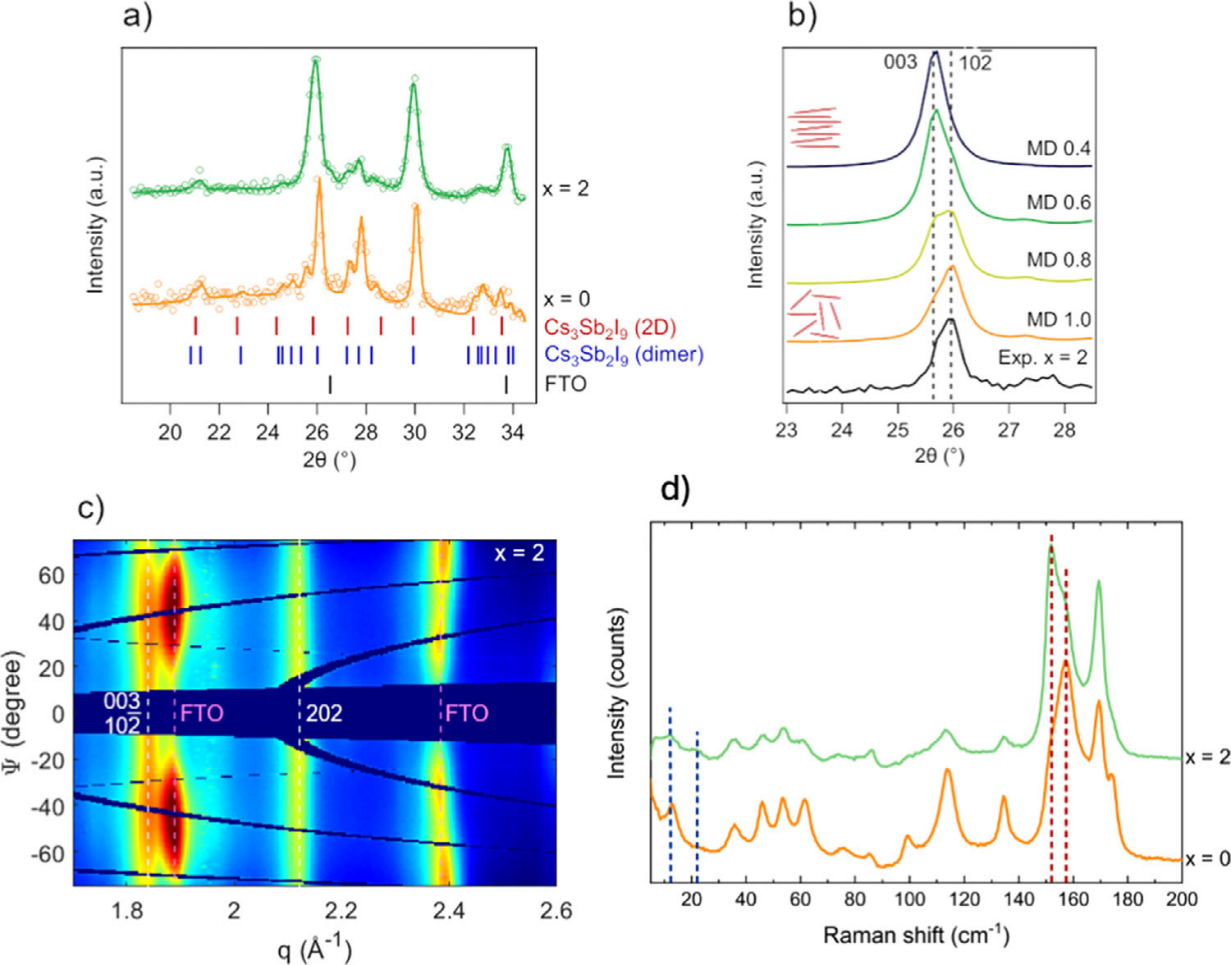
Structural analysis of Cs_3_Sb_2_I_9__−__x_Cl_x_ thin films. (a) Laboratory XRD patterns with Le Bail refinement highlighting the transition from the 0D dimeric polymorph to the layered 2D phase upon Cl incorporation. (b) Simulated preferential orientation effects using the March–Dollase model compared with the experimental pattern for *x* = 2. (c) GIWAXS polar map showing homogeneous diffraction rings of the layered phase, indicating the absence of strong preferential crystallographic orientation; (d) 80 K Raman spectra of Cs_3_Sb_2_I_9__−__x_Cl_x_ films deposited on Si for *x* = 0 and *x* = 2. Dashed lines mark the spectral regions most affected by Cl‐induced structural changes.

This conclusion is further supported by GIWAXS measurements. Although contributions from the FTO substrate are visible in the GIWAXS patterns, the absorber‐related reflections can be distinguished from the substrate features, allowing the crystallographic texture of the Cs_3_Sb_2_I_9__−__x_Cl_x_ phase to be assessed independently. The polar representation of the diffraction intensity (Figure 4c) reveals homogeneous diffraction rings associated with the layered phase, with no evidence of strong azimuthal modulation. Such behavior indicates the absence of pronounced preferential orientation of the perovskite crystallites across the investigated compositions. The additional GIWAXS maps reported in the Supplementary Information (Figure S15) confirm this trend for the entire series of films.

The absence of a strong crystallographic texture is particularly relevant when considering the potential impact of the layered structure on device operation. In layered vacancy‐ordered perovskites, charge transport is typically more favorable within the inorganic octahedral planes than perpendicular to them, and a strong orientation of these planes parallel to the substrate may therefore represent an unfavorable configuration for out‐of‐plane charge extraction [49, 50]. In the present case, XRD and GIWAXS consistently indicate that chlorine incorporation does not result in the development of such a strongly oriented layered texture. Thus, although these structural measurements do not directly probe carrier mobility or transport anisotropy, they show that the formation of the layered polymorph is not accompanied by the pronounced preferential orientation that could potentially impose an additional limitation on vertical charge extraction.

These observations provide a structural context for the improved photovoltaic response observed for the chlorine‐containing compositions. Rather than simply inducing a 0D‐to‐2D phase transition, chlorine acts as a phase‐fixing element, while the spray‐assisted processing route prevents the development of a strongly oriented layered texture. Together with the photophysical evidence of reduced carrier self‐trapping, these results are consistent with the improved device performance observed upon chlorine incorporation. However, a direct assessment of vertical versus lateral carrier mobility would be required to establish the relationship between crystallographic texture and transport anisotropy quantitatively.

To further probe the structural organization of the layered domains, low‐temperature Raman spectroscopy was performed. Measurements carried out at 80 K on Si substrates allow enhanced resolution of lattice vibrational modes and provide additional insight into the structural evolution induced by chlorine incorporation (Figure 4d), while Raman measurements on FTO substrates reproduce the same spectral trends and are reported in the Supplementary Information (Figure S16). In the pristine Cs_3_Sb_2_I_9_ films, characteristic vibrational features associated with the 0D dimeric phase are clearly observed, particularly in the 90–180 cm^−^^1^ region [30]. In contrast, the chlorine‐containing composition (*x* = 2) exhibits a marked suppression of these signatures, consistent with the dominant presence of the layered polymorph. Importantly, the ultralow frequency region (<30 cm^−^^1^) of the chlorine‐containing films reveals the emergence of distinct spectral features that are absent or less defined in the pristine material (blue dashed lines in Figure 4d). These modes can be associated with collective lattice vibrations involving interlayer interactions (as observed in 2D systems, e.g. graphene [60, 61], black phosphorous [62], and transition metal dichalcogenides [63]), indicating enhanced interlayer coupling within the layered domains (as recently shown for transition metal dichalcogenides samples where electric properties vary with stacking order [64]). Additional modifications in the 140–160 cm^−^^1^ region (red dashed lines in Figure 4d) further support a reorganization of the SbX_6_ octahedral framework consistent with the formation of a layered structure.

While Raman spectroscopy does not directly probe charge transport, these results provide complementary evidence that the chlorine‐induced layered phase is not only structurally present but also exhibits a vibrational signature consistent with coherent stacking and interlayer coupling. In combination with the GIWAXS analysis, this supports a picture in which the layered domains are distributed without a strongly detrimental texture and retain sufficient structural connectivity to enable effective out‐of‐plane charge extraction. This further corroborates the role of chlorine not only as a phase‐directing agent, but also as a key enabler of a device‐compatible microstructure in spray‐processed films.

## 3 Conclusions

We report an ambient‐air deposited and scalable route to lead‐free Cs_3_Sb_2_I_9__−__x_Cl_x_ absorbers by ultrasonic spray coating, showing how solvent engineering and halide compositional control can be coupled to enable efficient iPV operation. A low‐/medium‐boiling MethoxyEtOH/ButOH solvent system yields compact and uniform films under ambient conditions, overcoming a key processing bottleneck for antimony‐based perovskite‐inspired materials. At the same time, chloride incorporation does not merely alter composition, but directs annealing‐induced reconstruction from the dimeric 0D polymorph to a layered 2D phase. Combined XRD, GIWAXS, Raman, and transient spectroscopic analyses show that this transformation suppresses self‐trapping, improves band‐edge carrier persistence, and occurs without generating a strongly detrimental crystallographic texture, thereby preserving out‐of‐plane charge extraction despite the anisotropic layered structure.

These coupled structural and photophysical effects translate into clear device‐level benefits. Devices based on the optimized formulation and *x* = 2 composition reach an indoor PCE of 6.96% at 1000 lux under 6500 K illumination (stabilized iPCE = 6.5% @ MPP), together with improved operational stability, placing spray‐processed Cs_3_Sb_2_I_9_‐based absorbers in a competitive range for indoor energy harvesting. While the corresponding 1 sun performance remains limited, the present results clarify that the most relevant application space for these materials is low‐photon‐flux operation, where their bandgap, recombination landscape, and charge‐transport requirements are better matched to the working conditions.

More broadly, this work identifies a practical route to resolve the dimensionality‐transport trade‐off in lead‐free iPV s through scalable spray processing, chloride‐mediated dimensional reconstruction, and texture control within an ambient‐air deposition compatible process window. Beyond the specific Cs_3_Sb_2_I_9__−__x_Cl_x_ system, the results show that scalable deposition can be used not only to form lead‐free absorbers, but also to steer phase evolution and transport‐relevant microstructure in ways that are directly beneficial for vertical device operation.

## Author Contributions

**Francesco Lamberti**: conceptualization, funding acquisition, formal analysis, data curation, supervision, validation, methodology, writing – original draft, visualization, writing – review and editing. **Remah Elrashedy**: investigation, data curation. **Arpita Sarkar**: visualization, investigation, data curation. **Marcello Righetto**: conceptualization, investigation, formal analysis, validation, writing – original draft, writing – review and editing. **Ribhu Bhatia**: data curation, formal analysis, investigation, visualization, writing – original draft. **Pietro Ostellari**: data curation, investigation, visualization, writing – original draft. **Nicola Dengo**: data curation, visualization. **Mariangela Ruggeri**: data curation, investigation, validation, visualization. **Rosaria Verduci**: validation, investigation, visualization, data curation. **Andrea Basagni**: data curation, investigation. **Carlo Scian**: investigation. **Manas Ranjan Samantaray**: investigation, data curation. **Abhisek Chakraborty**: methodology, validation. **Zeynab Skafi**: methodology, validation. **Thomas Meredith Brown**: methodology, validation. **Guilin Liu**: formal analysis. **Jiacheng He**: data curation, investigation, visualization. **Zhubing He**: supervision. **Giovanna D'angelo**: validation, supervision. **Sara Pescetelli**: data curation, formal analysis, visualization, writing – review and editing, writing – original draft. **Camilla Ferrante**: supervision. **Simone Meloni**: conceptualization, data curation, funding acquisition, formal analysis, resources, supervision, software, methodology, visualization, project administration, writing – original draft, writing – review and editing, validation. **Teresa Gatti**: conceptualization, funding acquisition, supervision, resources, validation, methodology, writing – original draft, writing – review and editing, project administration. **Antonio Agresti**: conceptualization, formal analysis, funding acquisition, resources, supervision, validation, methodology, visualization, data curation, project administration, writing – review and editing, writing – original draft, investigation.

## Conflicts of Interest

The authors declare no conflicts of interests.

## Supporting information




**Supporting File**: smtd71072‐sup‐0001‐SuppMat.docx.

## Data Availability

All data supporting the findings of this study are provided within the Article and its Supplementary Information. Additional data are available from the corresponding authors upon reasonable request.
