# Supplementary material for: Scalable Spray Processing Resolves the Dimensionality–Transport Trade‐off in Lead‐Free Indoor Photovoltaics
Source: Small Methods. 2026 Sep 29;10(20):e71072. doi: 10.1002/smtd.71072 (PMC13706639; doi:10.1002/smtd.71072)
Supplement: Supplementary file 1 [file SMTD-10-e71072-s001.docx]

***Supporting Information for:***

**Scalable spray processing resolves the dimensionality–transport trade-off in lead-free indoor photovoltaics**

*F. Lamberti^1,2,3*^†, R. Elrashedy^1,4^†, A. Sarkar^5^, M. Righetto^1^, R. Bhatia^6^, P. Ostellari^1^, N. Dengo^1^, M. Ruggeri^7^, R. Verduci^7^, A. Basagni^1^, C. Scian^8^, M. Samantarai^5^, A. Chakraborty^5^, Z. Skafi^5^, T. M. Brown^5^, G. Liu^9^, J. He^10^, Z. He^10^, G. D’Angelo^7,11^, S. Pescetelli^5^, C. Ferrante^1^, S. Meloni^6*^, T. Gatti^4*^, A. Agresti^5*^*

^1^ Department of Chemical Science, Università degli Studi di Padova, Via Marzolo 1, 35131, Padova, Italy

^2^ Department of Information Engineering, Università degli Studi di Padova, Via Gradenigo 6/b, 35131, Padova, Italy

^3^ Zhejiang Beisheng Photovoltaic Co. Ltd., No 800 Zhenbei Road, Wuxing, Huzhou, Zhejiang 313000, China

^4^ Department of Applied Science and Technology, Politecnico di Torino, C.so Duca degli Abruzzi, 24, 10129, Torino, Italy

^5^ CHOSE, Centre for Hybrid Organic Solar Energy, Department of Electronic Engineering, University of Rome, Tor Vergata, Via del Politecnico 1, 000133, Roma, Italy

^6^ Dept. of Chemical and Pharmaceutical Sciences, Università of Ferrara, Via Luigi Borsari 46, 44121 Ferrara, Italy

^7^ Department of Mathematics and Computer Science, Physical Sciences and Earth Sciences, University of Messina, Viale F. Stagno d’Alcontres 31, S. Agata, 98166 Messina, Italy

^8^ Department of Physics and Astronomy, Università degli Studi di Padova, Via Marzolo 8, 35131, Padova

^9^ School of Optoelectronic Information and Physical Science, Jiangnan University, Wuxi 214122, China

^10^ Department of Materials Science and Engineering, Shenzhen Key Laboratory of Full Spectral Solar Electricity Generation (FSSEG), Institute of Major Scientific Facilities for New Materials, Southern University of Science and Technology (SUSTech), Shenzhen 518055, China

^11^ IMM-CNR sede di Messina, Viale Ferdinando Stagno d’Alcontres, 31, 98166 Messina, Italy

**Methods**

*Perovskites’ precursor solutions preparation.* Initially, to develop a greener, air-compatible processing route for lead-free Cs_3_Sb_2_I_9_ thin films, a systematic screening of four solvent systems (S1–S4; Table S1) has been conducted under ambient conditions. The precursor inks were prepared by combining separate stock solutions to precisely control solvation dynamics and ensure stoichiometric accuracy. CsI (Dyenamo, 99.99%) stock solutions (3 mM) has been prepared in pure ethanol, an ethanol/deionized water mixture (9:1 v/v), or 2-methoxyethanol. Whereas SbI_3_ (Sigma-Aldrich, 98%) stock solutions (2 mM) has been prepared in acetone or 1-butanol. All stock solutions were sonicated in ambient air for one hour to achieve complete dissolution. Consequently, the final Cs_3_Sb_2_I_9_ inks have been prepared by mixing the CsI and SbI_3_ stock solutions in a 1:1 volumetric ratio, achieving the target 3:2 Cs:Sb molar stoichiometry. The obtained precursor solution has been further sonicated for an additional 1 h. The S1–S4 systems have been selected for their reduced toxicity relative to conventional solvents, low-to-medium boiling points, and compatibility with ambient spray coating. Although 2-methoxyethanol is classified for reproductive toxicity (GHS Repr. 1B)^1^ the S4 system acts as a pragmatic compromise rather than a wholly benign medium. By eliminating DMF and chlorinated antisolvents while enabling ambient-air processing, S4 measurably reduces overall process hazards and provides the requisite rheological properties for scalable thin-film fabrication.

**Table S1:** Summary of the four engineered solvent systems screened for Cs_3_Sb_2_I_9_ precursor ink formulation.

| System | CsI Solvent | SbI_3_ Solvent |
| --- | --- | --- |
| S1 | Ethanol | Acetone |
| S2 | Ethanol | 1-Butanol |
| S3 | Ethanol / DI Water (9:1 v/v) | 1-Butanol |
| S4 | 2-Methoxyethanol | 1-Butanol |

Secondly, to improve the crystalline phase and film morphology, chloride has been introduced to synthesize Cs_3_Sb_2_I_9–x_Cl_x_ perovskites by utilizing SbCl_3_ (Sigma-Aldrich, 99%) as the chloride source while keeping a constant total antimony concentration of 2 mM. However, the relative ratio of SbI_3_ to SbCl_3_ has been systematically varied to target specific x values, which enables controlled investigation into the effects of halide substitution on the resulting thin films according to the following reaction:

However, it has been reported that excessive Cl substitution (x > 3.8) results in phase decomposition^2^, therefore, x-values have been selected within the range 0 to 3.0 to maintain structural stability. The composition with x = 0 corresponds to the reference Cs_3_Sb_2_I_9_ perovskite. Within this work, x refers to the nominal chlorine content in the precursor formulation and does not necessarily represent the chlorine stoichiometry of the final films.

*Thin-films fabrication.* Cs_3_Sb_2_I_9–x_Cl_x_ thin films were deposited by ultrasonic spray coating (ND-SP Ultrasonic Spray Coater, Nadetech Innovations, Spain) using the ultrasonic spray coating method, employing the previously prepared Cs_3_Sb_2_I_9–x_Cl_x_ precursor solutions, at relative humidity values between 40% and 20%. Prior to deposition, TiO_2_-coated FTO substrates were cleaned by UV-ozone treatment for 20 min to improve film adhesion. The substrates were placed on a hot plate set at 160 °C at a distance of 8 mm from the spray nozzle. The precursor solution was sprayed at a flow rate of 10 mL h^-1^ with a stage speed of 200 mm min⁻¹, resulting in films composed of five sequentially deposited layers. The as-deposited films were subsequently annealed gradually up to 250 °C on a hot plate in a closed chamber flushed with nitrogen, in the presence of SbI_3_ vapour.

*Device fabrication*. Mesoporous transparent titania paste (30 NR-D) is purchased from GreatCell Solar, while 2,20,7,70-tetrakis-(N,N-dip-methoxyphenylamine)9,9′-spirobifluorene (Spiro-MeOTAD) is from Borun**.** All other materials including lithium bis(trifluoromethanesulfonyl)imide (Li-TFSI), acetylacetone, ethanol, acetone, acetonitrile (ACN), tert-butylpyridine (tBP), chlorobenzene (CB), are purchased from Sigma-Aldrich. All materials are used as received unless specified otherwise. Patterned fluorine-doped tin oxide (FTO, Pilkington, 15 Ω □⁻¹) coated glasses were first cleaned with a detergent solution dissolved in deionized water and subsequently sonicated in acetone and ethanol for 10 minutes each. The compact TiO_2_ (c-TiO_2_) blocking layer (~40 nm) was deposited by spray pyrolysis at 460 °C and annealed for 30 minutes at the same temperature. The c-TiO_2_ precursor solution was composed of acetylacetone (2 mL), titanium isopropoxide (3 mL), and ethanol (45 mL). A mesoporous TiO_2_ (m-TiO_2_) scaffold layer was prepared from a commercial TiO_2_ paste (30 NR-D) diluted in ethanol (1:5 weight ratio), spin-coated at 3000 rpm for 20 s, and annealed in air for 30 minutes at 480 °C, yielding a ~120 nm thick porous layer. Subsequently the deposition of perovskite absorber layer (see above details for preparation and deposition), a spiro-OMeTAD solution (73.5 g·L^–1^ in chlorobenzene) doped with tert-butylpyridine (tBP), and lithium bis(trifluoromethanesulfonyl)imide (LiTFSI), was spin-coated at 3000 rpm for 20 s, forming a hole transport layer of approximately 140 nm. The LiTFSI precursor solution concentration was 520 mg·mL^–1^ in acetonitrile. Finally, the device was completed by thermal evaporation of a gold counter electrode (~100 nm) under high vacuum, defining an active area of 0.09 cm^2^ using a shadow mask.

*X-Ray diffraction (XRD) analysis and Grazing-Incidence Wide-Angle X-Ray Scattering (GIWAX).*

Grazing-incidence X-ray diffraction (GIXRD) measurements were carried out using a Bruker AXS D8 Advance Plus instrument equipped with a LYNEXEYE detector in 0D mode, by using Cu K_α1,2_ radiation (*λ*_1_ = 1.54060 Å; *λ*_2_ = 1.54439 Å, with relative intensity K_α2_/K_α1_ = 0.5). The incident beam was conditioned using a Göbel mirror to obtain a parallel beam geometry, and a 0.6 mm divergence slit was employed. The incident angle was fixed at 1.000° (θ) to enhance surface sensitivity. Diffraction patterns were collected over a 2θ range of 13-83° with a step size of 0.1° and a counting time of 8 s/step. Data analysis was performed using Bruker Diffrac.EVA software. Le Bail fiting was performed usign TOPAS-Academic Version 7^3^.

GIWAXS measurements were performed at the BL14B1 beamline of the Shanghai Synchrotron Radiation Facility. All measurements were performed at room temperature in air. The X-ray beam energy was set at 10.45 KeV (λ = 1.186 Å); the Pilatus 2 M detector was located 246.5 mm away from the sample stage. GIWAXS images were collected using a grazing incidence angle α = 0.3°. All GIWAXS images were analyzed using GDVis^4,5^.

*Temperature dependent Raman spectroscopy.* Micro-Raman spectroscopy measurements were performed in backscattering geometry using a HORIBA LabRam HR 800 and equipped with an ultralow-frequency filter (ULF) allowing acquisition of spectra down to ∼ 5 cm^−1^. A liquid nitrogen flow cryostat was used to control the sample temperature T = 80 K. A 532 nm laser was used for excitation, which was focused onto the sample surface using a 50x objective lens. The scattered light was dispersed by an 1800 gr/mm grating and detected using a liquid nitrogen cooled charge coupled device (CCD). The spectral resolution was 1 cm^−1^.

*UV-Visible spectroscopy.* The optical absorption spectra of the perovskite thin films were recorded in absorption mode with a spectrophotometer (CARY 50 Probe UV–vis spectrophotometer). The baseline was recorded using clean FTO.

*Scanning Electron Microscopy*. SEM was performed with a Zeiss Sigma HD microscope, equipped with a Schottky FEG source, one detector for backscattered electrons and two detectors for secondary electrons (InLens and Everhart Thornley). The microscope is coupled to an EDX detector (from Oxford Instruments, x-act PentaFET Precision) for X-rays microanalysis, working in energy dispersive mode. The cross-sectional images of PSCs were characterized by field-emission scanning electron microscopy (HITACHI SU8230).

*Transient Absorption (TA)*. TA measurements were performed using a home-built setup, described in detail elsewhere^6^. Briefly, pump and probe beams were obtained the fundamental output of an amplified Ti:Sapphire laser (SpitFire, Spectra Physics, 800 nm, 1kHz, 120 fs). The 400-nm pump beam was generated via second harmonic generation in a thin BBO thin crystal. The probe beam was converted into white-light probe pulses via supercontinuum white-light generation in a thin sapphire plate. To achieve a sufficient signal-to-noise ratio, the pump fluence was set to j = 60 µJ cm-2. The delay between pump and probe pulses was finely controlled with a linear motorised stage. The transmitted probe light and a reference beam were dispersed and directed to a linear CMOS diode array for detection.

*Profilometer analysis*. Film thickness was measured by stylus profilometry (Tencor P-17) using linear scans over 400 µm at a scan speed of 10 µm s⁻¹ and a sampling rate of 100 Hz. A stylus with 2 µm tip radius and an applied force of 0.5 mg was used. Measurements were performed with a 13 µm vertical range and 0.0078 Å resolution. Thickness values were extracted from rectangular step profiles obtained across a sharp film edge.

*Computational methodology*. Density functional calculations were performed within the Perdew-Burke-Ernzerhof (PBE) generalized gradient approximation as implemented in VASP code. The Kohn-Sham orbitals were expanded in a planewave basis set with an energy cutoff of 400 eV. The interaction between valence electron and nuclei plus core electron was described within the projector augmented wave (PAW) formalism. The Brillouin zone was sampled with 4 × 4 × 4 k-points mesh for the 2 × 2 × 2 bulk super-cells of 0D and 2D phases of Cs_3_Sb_2_I_9-x_Cl_x_. The relaxed 2D bulk super cells were then used to construct eight-layer thick slabs of Cs_3_Sb_2_I_9_ and Cs_3_Sb_2_I_7_Cl_2_ respectively, oriented along (100) direction. While experimental samples can have different surface terminations in a crystallite, our slab samples are symmetrically terminated. This is because computational samples are much smaller than experimental ones (few nanometers vs hundreds of nanometers to micrometers) and systematic asymmetries associated with different terminations can introduce a dipole moment in the slab, which may induce large electric fields. This likely induces artifacts in the computational results. Spiro-OMeTAD molecule was introduced only on one side of the slab, to obtain Cs_3_Sb_2_I_9-x_Cl_x_/Spiro-OMeTAD heterostructures. The heterostructures were modelled using periodic boundary conditions (PBC) in two directions (“b” and “c”, as shown in Figure S5a), while vacuum regions ~ 20 Å were introduced along surface normal (along direction “a”, see Figure S5a) on either side to prevent interactions between periodic images. The Cs_3_Sb_2_I_9-x_Cl_x_ slab samples were made thick enough to ensure that bulk like structural and electronic properties were in the central layers of the slab. Because the Spiro-OMeTAD molecule is placed only on one side of the slab, dipole corrections were also introduced during simulations. Finally, due to the large size of the heterostructure, gamma point sampling was employed to sample the Brillouin zone.

*Electro-optical measurements*: IPCE, ARKEO, I-V characteristics under 1 Sun and indoor conditions. Current–voltage (I–V) characteristics under 1 sun of masked devices were measured in ambient air using a class A solar simulator (ABET Sun 2000), calibrated to AM1.5G conditions at 100 mW cm^–2^ with a certified silicon reference cell (RERA Solutions RR-1002). The incident irradiance was monitored using a Skye SKS 1110 sensor, while the spectral match was verified with a BLACK-Comet UV–vis spectrometer. Both forward and reverse I–V scans were recorded, employing scan rates of 20 mV s⁻¹ for masked small-area PSCs (0.56 cm^2^ active area). The indoor PV measurement setup consisted of an enclosure with blackened interiors to minimize stray-light effects. Illumination was provided by an LED lamp (LEDVANCE WiFi SMART+ Dimmable White R50, 2700–6500 K) with a 60° beam angle. Measurements under different correlated colour temperatures (3000 K and 6500 K) and illuminance levels (200 lx and 1000 lx) were controlled via the LEDVANCE mobile application, which enabled adjustment of CCT and dimming. In accordance with IEC TS 62607-7-2:2023, the lamp exhibited Class SA temporal stability (after an initial stabilization period of ~5 min) and Class SA spatial uniformity over a 7 × 7 cm^2^ DUT area. The device under test (DUT) and sample holder were positioned approximately 50 cm from the lamp to improve spatial uniformity. To further suppress stray light, a black baffle plate was installed above the DUT. The spectral irradiance and incident power density at the DUT plane were measured using an Avantes AvaSpec-ULS2048CL-EVO spectroradiometer calibrated against a NIST-traceable irradiance standard. The illuminance at the DUT plane was measured with an HT 309 luxmeter, which complies with the general Class A specifications defined by JIS C 1609:1993 and CNS 5119. The indoor J–V measurements were performed over masked and not-encapsulated devices (0.1 cm^2^ active area) using a scan rate of 20 mV s⁻¹, with a voltage step of 10 mV and without any delay time, preconditioning or stabilization of the device before the J–V scan. The voltage scan was performed over the range reported in the corresponding J–V plots.

The illumination intensity dependence of Voc and dark I–V measurements were conducted using a modular characterization platform (Arkeo – Cicci Research s.r.l.) equipped with a white LED array (4200 K), adjustable up to 200 mW cm^–2^ optical power density, and a high-speed source meter unit operating at 600,000 samples s⁻¹ in a four-wire configuration. A spring-loaded contact sample holder was utilized to ensure improved measurement reproducibility.

Transient photovoltage (TPV) measurements were performed under large-perturbation conditions by recording the complete V_OC_ rise profiles following step increases in illumination intensity. Measurements were performed over a range of illumination intensities from 0.25 to 1 Sun under open-circuit conditions. Incident photon-to-current conversion efficiency (IPCE) spectra were acquired using an Arkeo system (Cicci Research s.r.l.) equipped with a 150 W xenon lamp and a double monochromator covering the 300–1400 nm range. Prior to IPCE measurements, the incident light intensity was calibrated using a silicon photodiode. Stability test under indoor condition were performed over encapsulated (Kapton tape + glass lid sealed with glass-glass Saratoga glue over the substrate frame) devices in ambient atmosphere (room temperature, 50% HR). During the light-soaking experiment, the device was continuously illuminated under 1000 lux (@6500) while maintained at open circuit. At regular time intervals (every 24 hours), the illumination was interrupted for 1-minute prior the J–V measurement, and a J–V curve was recorded under the same illumination conditions. Therefore, the light-soaking time refers to the cumulative illumination time excluding the short J-V measurement intervals. The iPCE reported in the stability plot was subsequently extracted from each corresponding J–V curve.

**Use of artificial intelligence tools**. The authors used ChatGPT (OpenAI) only for language editing and improvement of clarity during manuscript preparation. All scientific content, data analysis, interpretation, and conclusions were produced and validated by the authors. No artificial intelligence tool has been listed as an author.

**Supporting Results**

**Supporting optical characterization.**

**Figure S1**. Direct Tauc plots for MetoxyEtOH/ButOH films, showing E_g_ = 2.08 eV (as prepared) and 2.05 eV (annealed).

**Aging effect**. Building on this correlation between morphology and optical behaviour, Figure S1 examines how different solvent-derived films evolve when exposed to ambient air for 30 days. The temporal UV–Vis trends further reinforce the conclusions drawn from SEM and initial absorption measurements. Films prepared from Acetone/EtOH exhibit a progressive loss of optical density, consistent with their initially fragmented morphology, and show a clear broadening of the absorption edge over time. The ButOH/EtOH films, which already displayed the poorest continuity and the weakest absorbance, degrade most rapidly: after 10–15 days the spectrum is dominated by background signal, confirming the lack of structural integrity inferred from Figure 1c of the main manuscript. The H₂O/ButOH/EtOH films retain a recognizable absorption onset over the entire monitoring period, displaying only moderate intensity loss. This behaviour mirrors their partially continuous but locally heterogeneous morphology. In contrast, the MethoxyEtOH/ButOH films, identified as the most compact and homogeneous in the main text, demonstrate the highest environmental robustness: although a slight decrease in absorbance is observed, the spectral shape and band-edge region remain largely preserved after 30 days in air. Together, these ageing measurements show that films prepared with the MethoxyEtOH/ButOH formulation retain a well-defined absorption onset and a largely unchanged spectral shape after prolonged exposure to ambient air. This behaviour indicates a good optical robustness of the films, comparable to what is typically reported for non-encapsulated Cs₃Sb₂I₉ thin films obtained by conventional wet deposition methods^7^. In contrast, EtOH/ButOH films exhibit a rapid loss of spectral definition, consistent with their poor film continuity.

**Figure S2**. Time-dependent UV–Vis absorption spectra of Cs₃Sb₂I₉ films prepared from **(a)** Acetone/EtOH, **(b)** ButOH/EtOH, **(c)** H₂O/ButOH/EtOH, and **(d)** MethoxyEtOH/ButOH. Films were stored under ambient air and measured when fresh and after 10, 15, and 30 days. Differences in spectral evolution reflect the varying environmental stability of each solvent-derived morphology.

**Supporting morphological characterization**

**Figure S3.** XRD survey for Cs_3_Sb_2_Cl_x_I_9-x_ perovskites.

**Figure S4**. Top-view SEM images of the chloride-modified film, showing continuous coverage and densely packed grains; EDX ratios are reported (no detectable Cl signal).

**Supporting theoretical and spectroscopic analysis.**

We observed that in the Cs_3_Sb_2_I_9_/Spiro-OMeTAD heterostructure (Figure S5a-A/B) the latest occupied orbital near the interface has an energy lower than the corresponding band in the bulk of the system (Figure S5a-C). This implies that holes are pushed far from the interface, hindering or slowing down hole extraction. We hypothesize that incorporation of Cl- may alter the local electronic structure at Cs_3_Sb_2_I_9_/Spiro-OMeTAD interface, improving hole extraction. To test this hypothesis, we considered the interface between Spiro-OMeTAD and the absorbing layer presenting the best performance, Cs_3_Sb_2_I_7_Cl_2_. One notices that i) the energy difference between the bulk and surface valence maximum is reduced from 0.18 to 0.04 eV in going from Cs_3_Sb_2_I_9_ to Cs_3_Sb_2_I_7_Cl_2_. Similar to the previous analysis for the relative stability between 2D and 0D phases, while the effect of the incorporation of Cl^–^ in the structure on the energetics of bulk and interface electronic structure is apparently small, when compared with the thermal energy driving random walk of holes the “energy barrier” of approaching the HTM reduces from ~7 to ~1.5 the thermal energy at ambient conditions. In other words, while in Cs_3_Sb_2_I_9_ holes are strongly repelled from the interface with HTM, in Cs_3_Sb_2_I_7_Cl_2_ the energy to approach the Spiro-OMeTAD is closer to the thermal energy. Moreover, ii) since the inclusion of chloride has no effect on the electronic structure of Spiro-OMeTAD (Figure S5a-C/D), the shift of the valence band at the Cs_3_Sb_2_I_9-x_Cl_x_/Spiro-OMeTAD at higher energies increases the overlap of the energy between the local interface orbitals of the light harvesting material and the HTM, easing charge extraction.

**Figure S5**. **(a)** A) Cartoon illustration (side view) of Cs_3_Sb_2_I_9_ / Spiro-OMeTAD heterostructure used for DFT calculation; B) Shows the top view of the (100) surface slab onto which Spiro-OMeTAD was placed; C) pDOS for Cs_3_Sb_2_I_9_ / Spiro-OMeTAD heterostructure; D) pDOS for Cs_3_Sb_2_I_7_Cl_2_ / Spiro-OMeTAD heterostructure. The insets illustrate local valence band edge alignment of the bulk and surface layers; **(b)** Transient absorption spectroscopy on Cs_3_Sb_2_I_9–x_Cl_x_ films [x = 0, 2] processed using the MethoxyEtOH/ButOH formulation. A) Comparison between transient absorption spectra for x=0 (orange line) and x=2 (blue line) measured 10 ps following photoexcitation by a 400-nm pump pulse (30 uJ cm^–2^). B) Comparison between the bleaching recovery dynamics for x=0 (orange line) and x=1.5 (blue line) thin films. Dotted lines represent fit by a bi-exponential decay function.

To verify the effects of structural modification on the photophysics of Cs_3_Sb_2_I_9–x_Cl_x_, we employed ultrafast transient absorption (TA) spectroscopy. The TA spectra measured following high-energy (400 nm) photoexcitation are consistent with previous reports by Vivo and co-workers on Cs_3_Sb_2_I_9_ thin films^8,9^ (see Figure S6).

**Figure S6.** Transient absorption spectra of **(a)** x=0 Cs_3_Sb_2_Cl_x_I_9-_x **(b)** x=2 Cs_3_Sb_2_Cl_x_I_9-x_ thin films following measured 10 ps following photoexcitation by a 400-nm pump pulse (30 uJ cm^-2^). Early-time (< 5ps) transient absorption spectra of **(c)** x=0 Cs_3_Sb_2_Cl_x_I_9-x_ **(d)** x=2 Cs_3_Sb_2_Cl_x_I_9-x_ thin films following measured under the same photoexcitation conditions

The recorded spectra at fixed 10 ps time delay for both x=0 and x=2 (Figure S6b-A) feature three characteristic signals: a high-energy (< 500nm) ground-state bleaching signal, a broad photoinduced absorption (PIA) signal located at lower energies, and a weaker second bleaching signal at around 570nm, superimposed with the PIA. As previously demonstrated^8,9^, both bleaching features originate from electronic transitions in Cs_3_Sb_2_I_9_, whereas the broad PIA has been attributed to the formation and presence of self-trapped charge-carriers. Upon chloride incorporation (x=2), two clear differences emerge in the TA spectra. First, the GSB undergoes a redshift to about 500 nm, consistent with a slight bandgap narrowing associated with the 0D-to-2D phase transition, as indicated by XRD results. This is further supported by the band gaps of 0D (1.64) and 2D (1.53 eV) phases^10–12^ of Cs_3_Sb_2_I_7_Cl_2_, calculated using the slope of the total density of states (TDOS) curves, shown in Figure S7.

**Figure S7.** Total density of states (TDOS) used to compute band gaps of the 0D and 2D phases of Cs_3_Sb_2_I_7_Cl_2_.

Note that, while our DFT calculations underestimate the band gaps, a very well-known limitation^13^, it reliably captures relative band-gap trends. Secondly, we observe that the relative amplitude of the PIA signal, with respect to the main bleaching feature, is markedly reduced. The reduced prominence of this PIA signal indicates a decreased density of self-trapped charge-carriers upon chloride incorporation. This trend is further corroborated by the comparison of the bleaching recovery dynamics shown in Figure S6b-B. Such an early time dynamics has been previously attributed to the ultrafast self-trapping of free charge carriers^8,9^ (i.e., the small polaron formation dynamics) in Cs_3_Sb_2_I_9_. Therefore, the faster bleaching recovery dynamics observed for the x=0 sample (t_1/e = 2_ ps) suggest faster small-polaron formation, thereby confirming the enhanced self-trapping. These findings are fully consistent with the emerging role of structural and electronic dimensionality in inducing small polaron formation^14^. Here, we note that the predicted barrierless trapping for systems with low (e.g., 0D,1D) dimensionality is consistent with the faster dynamics observed for the x=0 film. Therefore, our findings further support observations on various perovskite-inspired materials, such as Cs_2_AgSbBr_6_, (AgI)_x_(BiI_3_)_y_, CuSbSe_2_^15–17^, and others, and highlight the role of dimensionality in emerging metal halides.

**Supporting device characterization**

**Supporting 1Sun characterization.** Under 1 sun illumination, statistical distributions of the key photovoltaic parameters, open circuit voltage (V_OC_), short circuit current density (J_SC_), fill factor (FF), and power conversion efficiency (PCE), (Figure S8(a–d) and Figure S9a-d; Tables S1-S2) reveal a systematic performance enhancement upon chlorine incorporation for both solvent systems, with a clear optimum at x=2. For devices processed using the MethoxyEtOH/ButOH formulation, the x=2 composition consistently delivers the highest efficiencies, with PCE values reaching ~ 1.62%, accompanied by Voc ≈ 0.66 V, Jsc ≈ 4.92 mA cm⁻², and FF ≈ 49.72% (Figure 4e, Table S1). In contrast, device fabricated using the ternary H₂O/ButOH/EtOH solvent system exhibit lower efficiencies (~1.18%), with reduced Voc ≈ 0.62 V, and Jsc ≈ 2.89 mA cm⁻² despite relatively high FF values (Figure S9e and Table S2). These differences are fully consistent with the structural and optical characteristics established earlier: the superior performance of the MethoxyEtOH/ButOH-based devices correlates with their more compact morphology, higher optical density, and increased fraction of layered stacking, as evidenced by SEM, UV–Vis, and XRD analyses.

External quantum efficiency (EQE) spectra for representative best –current devices (Figure S8f) further corroborate these trends. The integrated current densities derived from the EQE measurements closely match the Jsc values obtained from the J–V curves, confirming reliable current extraction and internal consistency of the photovoltaic characterization.

**Figure S8**. **(a-d)** Photovoltaic parameter statistics (8 samples, 0.56 cm^2^ active area) **a**) open circuit voltage (V_OC_); **b)** short circuit current density (J_SC_); **d)** fill factor (FF); **e)** power conversion efficiency (PCE), for the FTO/c-TiO_2_/m-TiO_2_/Cs₃Sb₂I₉₋ₓClₓ (x=0-3)/Spiro-OMeTAD/Au PISCs measured under 1 sun illumination, processed using the MethoxyEtOH/ButOH formulation. **c)** Current density-voltage (J-V) curves for the best performing Cs₃Sb₂I₉₋ₓClₓ (x=0, 2, MethoxyEtOH/ButOH ) PISCs measured acquired under 1 sun for both forward (empty symbol curves) and reverse (filled symbol curves) voltage scans. **f)** EQE and integrated current density for Cs₃Sb₂I₉₋ₓClₓ (x=0, 2, MethoxyEtOH/ButOH) PISCs.

**Table S2:** Photovoltaic parameters for the best PISCs with FTO/c-TiO_2_/m-TiO_2_/Cs₃Sb₂I₉₋ₓClₓ (x=0,2, H₂O/ButOH/EtOH)/Spiro-OMeTAD/Au structure, measured under 1 sun illumination.

| **1 Sun MethoxyEtOH/ButOH** | | | | |
| --- | --- | --- | --- | --- |
| **Device** | **V_OC_ (V)** | **J_SC_ (mA/cm^2^)** | **FF (%)** | **PCE (%)** |
| **x=2 (FW)** | 0.65 | 4.89 | 48.18 | 1.53 |
| **x=2 (RV)** | 0.66 | 4.92 | 49.72 | 1.62 |
| **x=0 (FW)** | 0.52 | 3.29 | 47.48 | 0.82 |
| **x=0 (RV)** | 0.52 | 3.13 | 53.52 | 0.88 |

**Figure S9**. **(a, b, d, e)** Photovoltaic parameter statistics (8 samples, 0.56 cm^2^ active area) for the FTO/c-TiO_2_/m-TiO_2_/Cs₃Sb₂I₉₋ₓClₓ (x=0-3)/Spiro-OMeTAD/Au PISCs measured under 1 sun illumination, processed using the H₂O/Butanol/Ethanol formulation. **c)** Current density-voltage (J-V) curves for the best performing Cs₃Sb₂I₉₋ₓClₓ (x=0, 2, H₂O/ButOH/EtOH) PISCs acquired under 1 sun for both forward (empty symbol curves) and reverse (filled symbol curves) voltage scans. **f)** EQE and integrated current density for Cs₃Sb₂I₉₋ₓClₓ (x=0, 2, H₂O/ButOH/EtOH) PISCs.

**Table S3:** Photovoltaic parameters for the best PISCs with FTO/c-TiO_2_/m-TiO_2_/Cs₃Sb₂I₉₋ₓClₓ (x=0-3, H₂O/ButOH/EtOH)/Spiro-OMeTAD/Au structure, measured under 1 sun illumination

| **1 Sun - H₂O/ButOH/EtOH** | | | | |
| --- | --- | --- | --- | --- |
| **Device** | **V_OC_ (V)** | **J_SC_ (mA/cm^2^)** | **FF (%)** | **PCE (%)** |
| **x=2 (FW)** | 0.61 | 3.07 | 61 | 1.14 |
| **x=2 (RV)** | 0.62 | 2.89 | 65.67 | 1.18 |
| **x=0 (FW)** | 0.54 | 1.02 | 55.06 | 0.30 |
| **x=0 (RV)** | 0.54 | 0.73 | 71.06 | 0.28 |

**Supporting Indoor characterization.**

**Figure S10.** **(a**): Maximum power point tracking (MPPT) measured under LED illumination (@ 1000 lux, 6500K) for the best performing Cs₃Sb₂I₉₋ₓClₓ (x=2) cell processed using the Methoxyethanol/Butanol solvent mixture. **(b)** Spectral irradiance and integrated Irradiance of the white LED (6500 K) used for the indoor measurements at 1000 and 200 lux.

**Table S4**: Photovoltaic parameters for the best devices of FTO/c-TiO_2/_m-TiO_2_/Cs₃Sb₂I₉₋ₓClₓ (x=0, 2, MethoxyEtOH/ButOH)/Spiro-OMeTAD/Au) perovskite-inspired solar cells measured @ 1000 lux, 6500K. The iPCE values are calculated by considering an integrated irradiance (Int. Irr.) of 338 μW/cm^2^.

| **1000 lux, 6500K - MethoxyEtOH/ButOH** | | | | |
| --- | --- | --- | --- | --- |
| **Device** | **V_OC_ (V)** | **J_SC_ (µA/cm^2^)** | **FF (%)** | **iPCE (%)** |
| **x=2 (FW)** | 0.50 | 65.3 | 63.33 | 6.15 |
| **x=2 (RV)** | 0.51 | 67.2 | 68.69 | 6.96 |
| **x=0 (FW)** | 0.29 | 41.2 | 53.87 | 1.90 |
| **x=0 (RV)** | 0.30 | 48.1 | 57.14 | 2.43 |

**Figure S11.** **(a-d)** Photovoltaic parameter statistics (8 samples, 0.1 cm^2^ active area) **a**) open circuit voltage (V_OC_); **b)** short circuit current density (J_SC_); **c)** fill factor (FF); **d)** power conversion efficiency (PCE), for the FTO/c- TiO_2_/m-TiO_2_/Cs₃Sb₂I₉₋ₓClₓ (=0 3)/Spiro-OMeTAD/Au PISCs measured @ 1000 lux, 6500K illumination, processed using the H₂O/ButOH/EtOH formulation. The iPCE values are calculated by considering an integrated irradiance (Int. Irr.) of 338 μW/cm^2^.

**Figure S12**. Current density-voltage (J-V) curve for the best performing Cs₃Sb₂I₉₋ₓClₓ (x=0, 2) processed using H₂O/Butanol/Ethanol) formulations for PISCs measured at 1000 lux and 6500K.

**Table S5**: Photovoltaic parameters for the best PISCs with FTO/c-TiO_2_/m-TiO_2_/Cs₃Sb₂I₉₋ₓClₓ (x = 0, 2, H₂O/ButOH/EtOH)/Spiro-OMeTAD/Au structure, measured @ 1000 lux, 6500K. The iPCE values are calculated by considering an integrated irradiance (Int. Irr.) of 338 μW/cm^2^.

| **1000 lux, 6500K - H₂O/ButOH/EtOH** | | | | |
| --- | --- | --- | --- | --- |
| **Device** | **V_OC_ (V)** | **J_SC_ (µA/cm^2^)** | **FF (%)** | **iPCE (%)** |
| **x=2 (FW)** | 0.50 | 39.7 | 60.51 | 3.55 |
| **x=2 (RV)** | 0.50 | 42.5 | 63 | 3.96 |
| **x=0 (FW)** | 0.37 | 23.9 | 54.01 | 1.41 |
| **x=0 (RV)** | 0.37 | 26.3 | 55.02 | 1.58 |

**Figure S13**: **(a-d)** Photovoltaic parameter statistics (8 samples, 0.1 cm^2^ active area) **a**) open circuit voltage (V_OC_); **b)** short circuit current density (J_SC_); **c)** fill factor (FF); **d)** power conversion efficiency (PCE), for the FTO/c-TiO_2_/m-TiO_2_/Cs₃Sb₂I₉₋ₓClₓ (x=0-3)/Spiro-OMeTAD/Au PISCs measured @ 200 lux, 6500K illumination, processed using the MethoxyEtOH/ButOH formulation. The iPCE values are calculated by considering an integrated irradiance (Int. Irr.) of 75 μW/cm^2^.

**Supporting Electro-optical characterization.**

**Figure S14**. **a)** Nyquist plots, **b)** Dark J−V and **c)** photovoltage rise time (τ_rise_) vs voltage curves for PISCs with FTO/c-TiO_2_/m-TiO_2_/Cs₃Sb₂I₉₋ₓClₓ (x=0 and x=2, MethoxyEtOH/ButOH)/Spiro-OMeTAD/Au structure.

**Supporting GIWAX characterization**

**Figure S15**. GIWAXS 2D images in q-space (top) and polar space (down) for Cs_3_Sb_2_Cl_x_I_9-x_ films prepared with different amounts of Cl precursors. The patterns for x = 2 and 3 are dominated by the contributions of the underlying layer of FTO, showing preferential orientation, while the pattern from Cs_3_Sb_2_Cl_x_I_9-x_ shows no evident orientation (i.e., crystallites are randomly oriented).

**Supporting Raman characterization**

**Figure S16**. 80 K Raman spectra of Cs₃Sb₂I₉₋ₓClₓ films deposited on FTO for x = 0 and x = 2. Dashed lines mark the spectral regions most affected by Cl-induced structural changes.

**Supporting Bibliography**

(1)	European Chemical Agency. 2-Methoxyethanol – Harmonised Classification and Labelling (CLP Regulation) https://echa.europa.eu/it/substance-information/-/substanceinfo/100.003.377 (accessed Feb 3, 2026).

(2)	Paul, G.; Pal, A. J.; Larson, B. W. Structure, Morphology, and Photovoltaic Implications of Halide Alloying in Lead‐Free Cs 3 Sb 2 Cl x I 9– x 2D‐Layered Perovskites. *Sol. RRL* **2021**, *5* (1). https://doi.org/10.1002/solr.202000422.

(3)	Coelho, A. A. TOPAS and TOPAS-Academic : An Optimization Program Integrating Computer Algebra and Crystallographic Objects Written in C++. *J. Appl. Crystallogr.* **2018**, *51* (1), 210–218. https://doi.org/10.1107/S1600576718000183.

(4)	Schrode, B.; Pachmajer, S.; Dohr, M.; Röthel, C.; Domke, J.; Fritz, T.; Resel, R.; Werzer, O. GIDVis : A Comprehensive Software Tool for Geometry-Independent Grazing-Incidence X-Ray Diffraction Data Analysis and Pole-Figure Calculations. *J. Appl. Crystallogr.* **2019**, *52* (3), 683–689. https://doi.org/10.1107/S1600576719004485.

(5)	Ashiotis, G.; Deschildre, A.; Nawaz, Z.; Wright, J. P.; Karkoulis, D.; Picca, F. E.; Kieffer, J. The Fast Azimuthal Integration Python Library: PyFAI. *J. Appl. Crystallogr.* **2015**, *48* (2), 510–519. https://doi.org/10.1107/S1600576715004306.

(6)	Righetto, M.; Privitera, A.; Carraro, F.; Bolzonello, L.; Ferrante, C.; Franco, L.; Bozio, R. Engineering Interactions in QDs–PCBM Blends: A Surface Chemistry Approach. *Nanoscale* **2018**, *10* (25), 11913–11922. https://doi.org/10.1039/C8NR03520B.

(7)	Park, Y.; Kim, S. H.; Lee, D.; Lee, J.-S. Designing Zero-Dimensional Dimer-Type All-Inorganic Perovskites for Ultra-Fast Switching Memory. *Nat. Commun.* **2021**, *12* (1), 3527. https://doi.org/10.1038/s41467-021-23871-w.

(8)	Lamminen, N.; Grandhi, G. K.; Fasulo, F.; Hiltunen, A.; Pasanen, H.; Liu, M.; Al‐Anesi, B.; Efimov, A.; Ali‐Löytty, H.; Lahtonen, K.; et al. Triple A‐Site Cation Mixing in 2D Perovskite‐Inspired Antimony Halide Absorbers for Efficient Indoor Photovoltaics. *Adv. Energy Mater.* **2023**, *13* (4). https://doi.org/10.1002/aenm.202203175.

(9)	Lamminen, N.; Karlsson, J.; Kumar, R.; Viswanath, N. S. M.; Lal, S.; Fasulo, F.; Righetto, M.; Krishnaiah, M.; Lahtonen, K.; Tewari, A.; et al. The Promise of Operational Stability in Pnictogen-Based Perovskite-Inspired Solar Cells. *EES Sol.* **2025**, *1* (2), 139–156. https://doi.org/10.1039/D5EL00029G.

(10)	Saparov, B.; Hong, F.; Sun, J.-P.; Duan, H.-S.; Meng, W.; Cameron, S.; Hill, I. G.; Yan, Y.; Mitzi, D. B. Thin-Film Preparation and Characterization of Cs 3 Sb 2 I 9 : A Lead-Free Layered Perovskite Semiconductor. *Chem. Mater.* **2015**, *27* (16), 5622–5632. https://doi.org/10.1021/acs.chemmater.5b01989.

(11)	Jiang, F.; Yang, D.; Jiang, Y.; Liu, T.; Zhao, X.; Ming, Y.; Luo, B.; Qin, F.; Fan, J.; Han, H.; et al. Chlorine-Incorporation-Induced Formation of the Layered Phase for Antimony-Based Lead-Free Perovskite Solar Cells. *J. Am. Chem. Soc.* **2018**, *140* (3), 1019–1027. https://doi.org/10.1021/jacs.7b10739.

(12)	Exner, A.; Gouvêa, R. A.; Köche, A.; Khan, S.; Santos, J. F. L.; José Leite Santos, M. Doping Effects on the Optoelectronic Properties and the Stability of Cs3Sb2I9: Density Functional Theory Insights on Photovoltaics and Light-Emitting Devices. *J. Sci. Adv. Mater. Devices* **2024**, *9* (2), 100700. https://doi.org/10.1016/j.jsamd.2024.100700.

(13)	Mori-Sánchez, P.; Cohen, A. J.; Yang, W. Localization and Delocalization Errors in Density Functional Theory and Implications for Band-Gap Prediction. *Phys. Rev. Lett.* **2008**, *100* (14), 146401. https://doi.org/10.1103/PhysRevLett.100.146401.

(14)	Buizza, L. R. V.; Herz, L. M. Polarons and Charge Localization in Metal‐Halide Semiconductors for Photovoltaic and Light‐Emitting Devices. *Adv. Mater.* **2021**, *33* (24). https://doi.org/10.1002/adma.202007057.

(15)	Lal, S.; Righetto, M.; Putland, B. W. J.; Sansom, H. C.; Motti, S. G.; Jin, H.; Johnston, M. B.; Snaith, H. J.; Herz, L. M. The Role of Chemical Composition in Determining the Charge‐Carrier Dynamics in (AgI)x(BiI3)y Rudorffites. *Adv. Funct. Mater.* **2024**, *34* (32). https://doi.org/10.1002/adfm.202315942.

(16)	Righetto, M.; Caicedo-Dávila, S.; Sirtl, M. T.; Lim, V. J.-Y.; Patel, J. B.; Egger, D. A.; Bein, T.; Herz, L. M. Alloying Effects on Charge-Carrier Transport in Silver–Bismuth Double Perovskites. *J. Phys. Chem. Lett.* **2023**, *14* (46), 10340–10347. https://doi.org/10.1021/acs.jpclett.3c02750.

(17)	Fu, Y.; Lohan, H.; Righetto, M.; Huang, Y.-T.; Kavanagh, S. R.; Cho, C.-W.; Zelewski, S. J.; Woo, Y. W.; Demetriou, H.; McLachlan, M. A.; et al. Structural and Electronic Features Enabling Delocalized Charge-Carriers in CuSbSe2. *Nat. Commun.* **2025**, *16* (1), 65. https://doi.org/10.1038/s41467-024-55254-2.
